# Value of shear wave arrival time contour display in shear wave elastography for breast masses diagnosis

**DOI:** 10.1038/s41598-017-07389-0

**Published:** 2017-08-01

**Authors:** Bang-Guo Zhou, Dan Wang, Wei-Wei Ren, Xiao-Long Li, Ya-Ping He, Bo-Ji Liu, Qiao Wang, Shi-Gao Chen, Azra Alizad, Hui-Xiong Xu

**Affiliations:** 1 Department of Medical Ultrasound, Shanghai Tenth People’s Hospital, Ultrasound Research and Education Institute, Tongji University School of Medicine, Shanghai, 200072 China; 2 Thyroid Institute, Tongji University School of Medicine, Shanghai, 200072 China; 3 Department of Radiology, Mayo Clinic, 200 First Street SW, Rochester, Minnesota 55905 USA

## Abstract

To evaluate the diagnostic performance of shear wave arrival time contour (SWATC) display for the diagnosis of breast lesions and to identify factors associated with the quality of shear wave propagation (QSWP) in breast lesions. This study included 277 pathologically confirmed breast lesions. Conventional B-mode ultrasound characteristics and shear wave elastography parameters were computed. Using the SWATC display, the QSWP of each lesion was assigned to a two-point scale: score 1 (low quality) and score 2 (high quality). Binary logistic regression analysis was performed to identify factors associated with QSWP. The area under the receiver operating characteristic curve (AUROC) for QSWP to differentiate benign from malignant lesions was 0.913, with a sensitivity of 91.9%, a specificity of 90.7%, a positive predictive value (PPV) of 74.0%, and a negative predictive value (NPV) of 97.5%. Compared with using the standard deviation of shear wave speed (SWS_SD_) alone, SWS_SD_ combined with QSWP increased the sensitivity from 75.8% to 93.5%, but decreased the specificity from 95.8% to 89.3% (P < 0.05). SWS_SD_ was identified to be the strongest factor associated with the QSWP, followed by tumor malignancy and the depth of the lesion. In conclusion, SWATC display may be useful for characterization of breast lesions.

## Introduction

It has been noted that breast cancer is stiffer than normal breast tissue and the stiffening process begins in the early stage of cancer. Therefore, ultrasound elastography is often used to help diagnose breast lesions. There are two types of elastography technologies: strain elastography and shear wave elastography^1^. Strain elastography provides a map of tissue deformation when the lesion is manually compressed by the ultrasound transducer. Shear wave elastography assesses the speed (V, which is related to the Young modulus in kilopascals by 3V^2^) of shear wave propagation within the lesion^2^. Strain elastography typically can only provide qualitative or semi-quantitative information and is more operator-dependent. Shear wave elastography provides quantitative information of tissue stiffness, and is generally less operator dependent and more reproducible^3, 4^. Therefore, shear wave elastography is used more often as a supplement to conventional ultrasound imaging in clinical practice.

Shear wave elastography has been shown to improve the diagnostic performance in differentiating benign from malignant breast lesions^5, 6^. However, it has been noted that low quality of shear wave propagation (QSWP) detected in the tissue may lead to invalid shear wave speed measurements^7, 8^. For example, shear wave measurements in simple cysts are often invalid because shear waves cannot propagate in liquid. As another example, shear wave elastography in invasive cancers typically have a higher failure rate because shear wave measurements in very stiff lesions are often unreliable^9^. The QSWP may also be influenced by the transducer motion, patient motion, lesion depth, tissue inhomogeneity, calcifications^10^ etc., which may lead to incorrect measurements in some lesions.

Several ultrasound companies have provided tools to help users determine if a shear wave measurement is reliable or not. In virtual touch quantification (VTQ; Siemens Medical Solutions, Mountain View, CA, USA), a 2D quality map is provided where the green color represents high quality for shear wave speed measurement while yellow or red color indicates low quality. For Supersonic Imagine (SSI, Aix-en-Provence, France), regions with low QSWP are not color-coded. Toshiba scanners (Toshiba Medical System, Tochigi, Japan) provide a “propagation mode” that displays the shear wave arrival time contours (SWATC) to help users evaluate the reliability of shear wave measurements. The intervals between the displayed contour lines are wider in stiff tissues and narrower in soft tissues. In areas where the contour lines are parallel, the shear waves propagate properly and the reliability of the obtained data is high. On the contrary, in areas where the contour lines are distorted and not parallel to one another, the reliability of the obtained data is low. These quality assurance tools are useful for users to select regions of high shear wave measurement confidence to increase the reliability of measurements.

Barr *et al*.^8^ recently found that the addition of a quality measurement of shear wave speed estimation can increase sensitivity (from 50% to 93%) for breast cancer detection without significant loss of specificity (from 94% to 89%). They believed that low quality measurement might be a feature of malignancy. However, the low QSWP may also be observed in some benign breast lesions^7^. Thus, it is important to investigate factors associated with the QSWP, which can help to identify and disentangle these confounding factors and improve the accuracy of diagnosis. Our study thus aims to evaluate the diagnostic performance of SWATC display for the diagnosis of breast lesions, and to identify factors associated with the QSWP in breast lesions.

## Materials and Methods

### Patients

This retrospective study was approved by The Ethical Committee of Shanghai Tenth People’s Hospital. Due to the retrospective nature of the study, the requirement to obtain informed consent from the patients was waived. This study was performed in accordance with the Declaration of Helsinki for human study. From January 2016 to July 2016, seven hundred and seventeen consecutive patients with suspicious breast lesions had conventional ultrasound examination and shear wave elastography. The inclusion criteria were: (a) no history of treatment such as surgery, radiotherapy, or chemotherapy before ultrasound examination; (b) with histopathologic findings; (c) breast lesions can be detected by ultrasound; (d) solid breast lesions or approximate solid lesions (<25% cystic). A total of 288 breast lesions in 284 patients met the criteria. For patients with more than one lesion, the lesion with the highest ultrasound Breast Imaging Reporting and Data System (BIRADS) category was chosen. If there were multiple lesions with the same highest BI-RADS category, all of them were chosen. Among these 284 patients, 11 patients had incomplete data and were excluded. Finally, a total of 277 breast masses (215 benign, 62 malignant) in 273 patients (mean age 45.1 ± 14.6 years; range 15–85 years) were included in this study. The mean lesion size on B-mode ultrasound measurement was 15.6 ± 8.5 mm (range, 4.1–63.2 mm).

### Ultrasound Examination

Conventional ultrasound and shear wave elastography examinations were performed using the same Aplio500 ultrasound scanner (Toshiba Medical Systems Corporation, Tochigi, Japan) with a 14L5 liner array transducer (frequency range, 5–14 MHz), by one of two board-certified radiologists with more than 2-years of experience in breast ultrasound and elastography. For conventional ultrasound, a standard scanning protocol was used to obtain both transverse and longitudinal images of each target lesion^11^. Shape (oval/round, irregular), orientation (parallel, not parallel to skin), margin (circumscribed, non-circumscribed), lesion depth (measured as the distance from the skin to the center of the mass), echo pattern (isoechoic, complex cystic and solid, hypoechoic etc.), posterior features (unchanged, changed), calcifications (present, absent), lesion size (maximal diameter as measured on ultrasound) and vascularity (present, absent) on color Doppler images were recorded. Afterwards, Lesions were classified according to the ultrasound BI-RADS lexicon of American College of Radiology (ACR)^12^.

### Shear Wave Elastography

**Figure 1:**
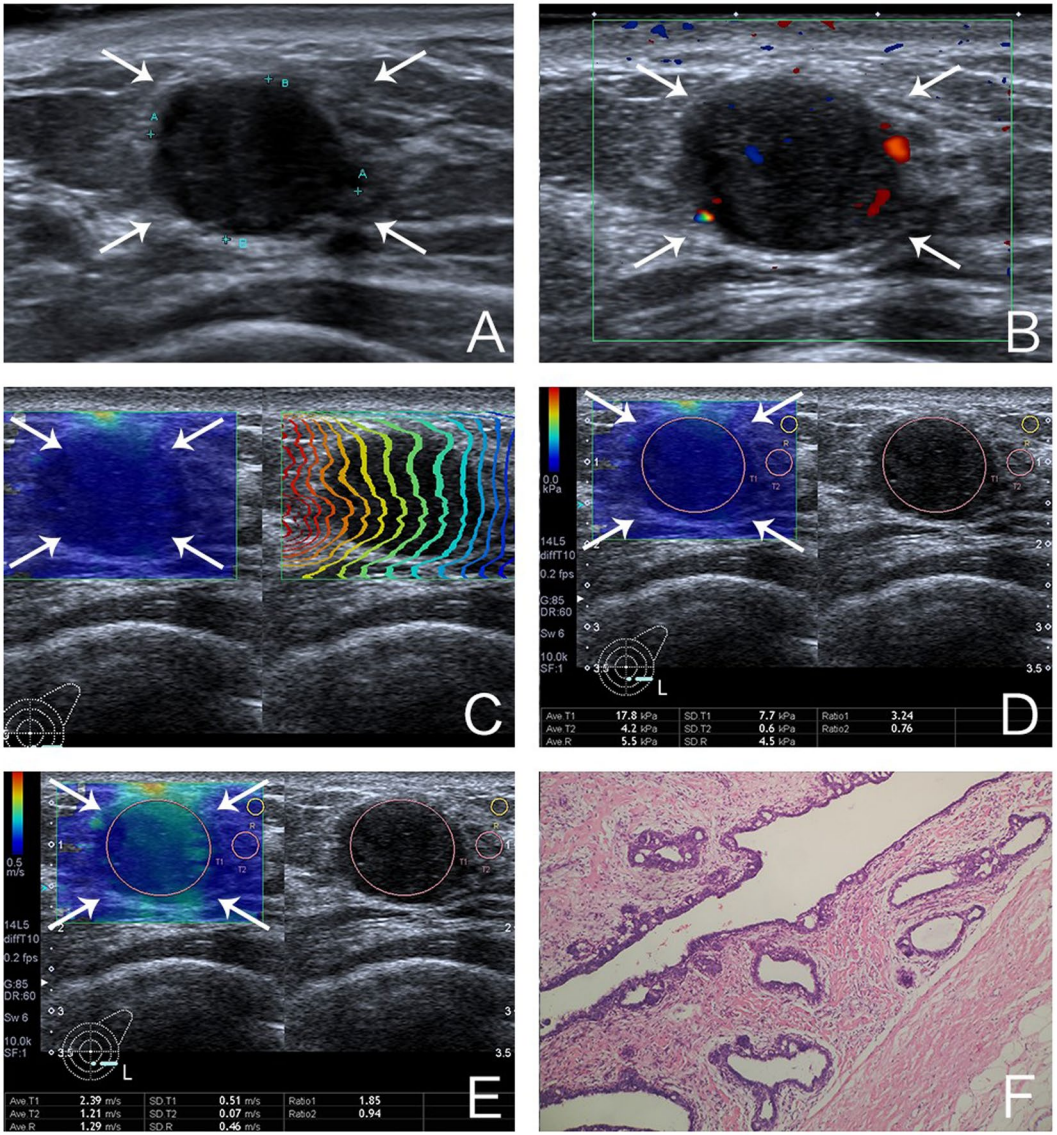
Images of a 31-year-old woman with fibroadenoma. (**A**) The lesion is shown on B-mode ultrasound. (**B**) The lesion is shown on color Doppler ultrasound (**C**) shear wave arrival time contour of the lesion shows regularly parallel lines on the shear wave propagation mode. (**D**) The mean and standard deviation of the lesion on elasticity mode are 17.8 kPa and 7.7 kPa, respectively. (**E**) The mean and standard deviation of the lesion on shear wave speed mode are 2.39 m/s and 0.51 m/s, respectively. (**F**) Pathological examination confirms the diagnosis of fibroadenoma (Hematoxylin-eosin stain; × 100).

**Figure 2:**
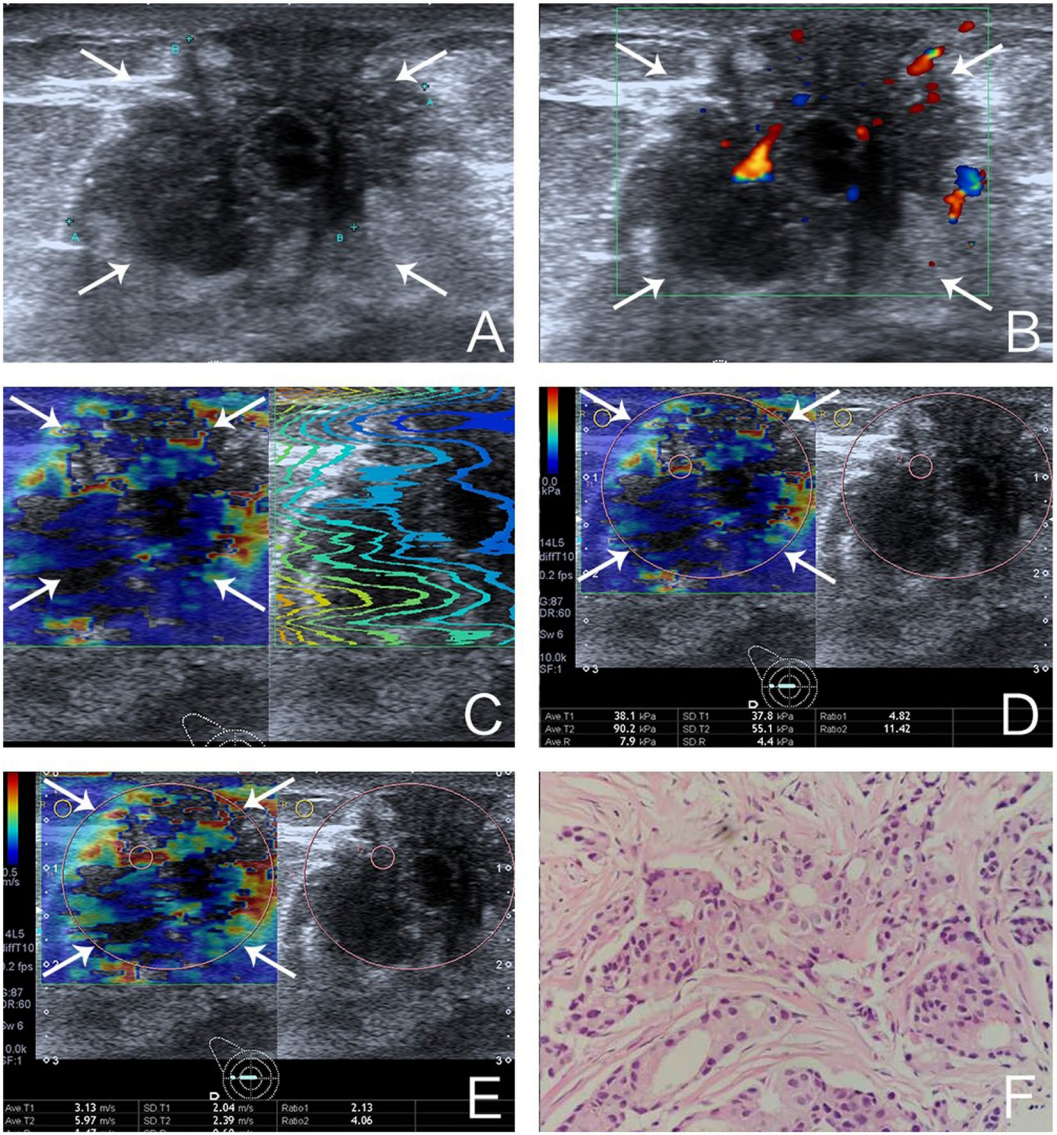
Images of a 57-year-old woman with invasive ductal carcinoma. (**A**) The lesion is shown on B-mode ultrasound. (**B**) The lesion is shown on color Doppler ultrasound (**C**) shear wave arrival time contour of the lesion shows distorted and unparalleled lines on the shear wave propagation mode. (**D**) The mean and standard deviation of the lesion on elasticity mode are 38.1 kPa and 37.8 kPa, respectively. (**E**) The mean and standard deviation of the lesion on shear wave speed mode are 3.13 m/s and 2.04 m/s, respectively. (**F**) Pathological examination confirms the diagnosis of invasive ductal carcinoma (Hematoxylin-eosin stain; × 200).

**Figure 3:**
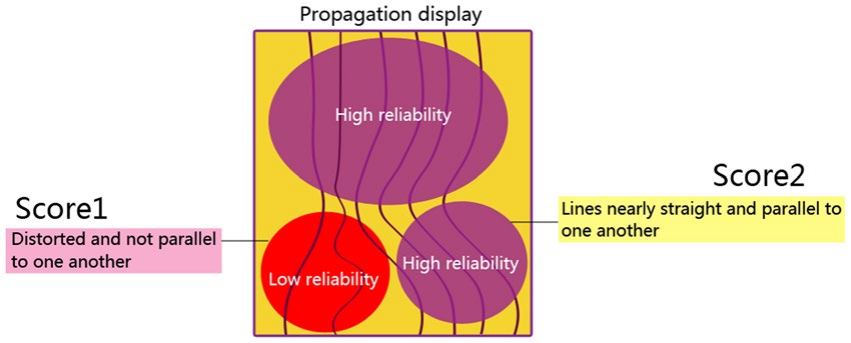
Image explanation for the discrepancy of quality scores for determination. Score1 (low quality) was assigned when the contour lines are distorted and unparalleled; score 2 (high quality) was assigned to lesions with parallel lines.

Shear wave elastography measurements were obtained after conventional ultrasound imaging by the same operator. When obtaining shear wave elastography, patients were asked to suspend respiration for several seconds. The transducer was kept perpendicular to the body surface with minimal compression because excessive compression can change the stiffness of tissue. The lesion of interest was placed in the center of the ultrasound image. After the ultrasound image was optimized, the “one shot scan” mode in which image quality is given higher priority was selected to acquire the shear wave image (Figs 1 and 2). There are three options to display data after imaging frozen: elasticity mode, propagation mode, and speed mode. The QSWP was assessed using a two-point scale based on the shape of the contour lines displayed in the propagation mode. Score1 (low quality) was assigned when the contour lines are distorted and unparalleled; score 2 (high quality) was assigned to lesions with parallel lines (Fig. 3). Subsequently, elasticity and speed mode were successively selected, the region of interest (ROI) was artificially set to cover the lesions. The size of ROI can be adjusted according to the shape of the target lesion in both elasticity and speed mode. To ensure the reliability of SWE, distorting factors such as calcification, obvious cystic parts or surrounding tissue of breast lesions were avoided when placing the ROI box on the image. For lesions with low quality of shear wave propagation, two ROI boxes were selected. One ROI was adjusted according to the lesion shape to encompass the maximum lesion area to acquire the E-mean, E_SD_, SWS-mean and SWS_SD_ of the lesion. The other was placed on the stiffest area to obtain the maximum value of elastic and speed according to the color map on which stiff tissues were coded with red, with areas of decreasing stiffness coded with orange, green, light blue, and dark blue (Fig. 2). And then the scanner automatically calculated the mean elasticity (E-mean), elasticity standard deviation (E_SD_), mean shear wave speed (SWS-mean) and standard deviation of shear wave speed (SWS_SD_).

### Image Interpretation

Two radiologists with more than 2 years of experiences in shear wave elastography reviewed the images to choose the image which quality is best and the propagation contour map to assign a quality score for each lesion. In case of discrepancies, consensus was obtained by consulting with a third supervising radiologist. All radiologists were blinded to patient data including clinical information and grayscale images.

### Histologic Diagnosis

Patients had ultrasound-guided breast biopsy (at least three samples obtained) or surgical removal of the lesion to obtain histopathological readings for comparison with ultrasound results.

### Statistical Analysis

All statistical analyses were performed using the SPSS software (version 20.0; SPSS, Chicago, III). Mean ± standard deviation was calculated for continuous data with normal distribution, and the difference was compared using t test. χ2 test or Fisher’s exact probability test was used to compare categorical variables. A statistically significant difference was defined as P < 0.05. Main statistical analysis was composed of two parts. First, diagnosis performance of different shear wave elastography parameters (E-mean, E_SD_, SWS-mean, SWS_SD_, and QSWP) was evaluated. The t test was used to investigate the difference between benign and malignant lesions. With histopathologic diagnosis as the reference standard, the diagnostic performances for all shear wave elastography parameters were evaluated by receiver operating characteristic (ROC) curve analysis. Sensitivity, specificity, negative predictive value (NPV), positive predictive value (PPV), and area under ROC curve (AUROC) were calculated. The cut-off value of each parameter was selected when the Youden index (sensitivity + specifcity-1) reached the maximum value. The comparisons of sensitivity and specificity for different parameters were performed using the McNemar test. Second, binary logistic regression was used to identify factors associated with the QSWP. All ultrasound parameters showing a significant difference between high and low QSWP were used in the binary logistic regression.

## Results

### Basic characteristics

There were 215 (78%) benign and 62 (22%) malignant lesions (Table 1) in this study. The mean age of patients with malignant breast lesions (59.7 ± 12.8 years; range: 31–85 years) was significantly higher than the age of patients with benign breast lesions (40.9 ± 12.2 years; range: 15–81 years). The maximum diameter of malignant breast lesions (20.5 ± 9.5 mm; range: 7.0–61.9 mm) was significantly higher than that of benign lesions (14.2 ± 7.7 mm; range: 4.1–63.2 mm). For conventional ultrasound features, irregular shape, non-parallel orientation, changed posterior features (post lesion enhancement, shadowing, or their combination), non-circumscribed margin, and calcification were more commonly found in malignant breast lesions (all *P* < 0.05) (Table 2).

**Table 1:** Final histologic features of breast lesions.

Pathology	NO. of lesions
Benign	215
Fibroadenoma	133
Adenosis	71
Intraductal papilloma	4
Benign phyllodes tumor	3
Tubular adenoma	1
Inflammatory granulation	1
Subacute inflammatory fibrous hyperplasia	1
Fibrofatty tissue	1
Malignant	62
Invasive ductal carcinoma	52
Intraductal carcinoma	5
Mucinous carcinoma	3
Invasive lobular carcinoma	1
Apocrine carcinoma	1

**Table 2:** Ultrasound and shear wave elastography characteristics of benign and malignant lesions.

Characteristic	Overall	Malignant	Benign	*P*-value
Patients	277	62	215	
		(22%)	(78%)	
Mean age (year)	45.1 ± 14.6	59.7 ± 12.8	40.9 ± 12.2	<0.001*
Lesions				
Diameter (mm)	15.6 ± 8.5	20.5 ± 9.5	14.2 ± 7.7	<0.001*
Shape				<0.001*
Oval/Round	158	12	146	
	(57.0%)	(19.4%)	(67.9%)	
Irregular	119	50	69	
	(43.0%)	(80.6%)	(32.1%)	
Lesion depth				<0.001*
>15mm	90	39	51	
	(32.5%)	(62.9%)	(23.7%)	
<15mm	187	23	164	
	(67.5%)	(37.1%)	(76.3%)	
Orientation				<0.001*
Parallel	222	38	184	
	(80.1%)	(61.3%)	(85.6%)	
Not parallel	55	24	31	
	(19.9%)	(38.7%)	(14.4%)	
Margin				<0.001*
Circumscribed	161	14	147	
	(58.1%)	(22.6%)	(68.4%)	
Non-circumscribed	116	48	68	
	(41.9%)	(77.4%)	(31.6%)	
Posterior features				<0.001*
Changed	44	19	25	
	(15.9%)	(30.6%)	(11.6%)	
Unchanged	233	43	190	
	(84.1%)	(69.4%)	(88.4%)	
Calcifications				<0.001*
Present	34	16	18	
	(12.3%)	(25.8%)	(8.4%)	
Absent	243	46	197	
	(87.7%)	(74.2%)	(91.6%)	
Vascularity				<0.001*
Present	71	38	33	
	(25.6%)	(61.3%)	(15.3%)	
Absent	206	24	182	
	(74.4%)	(38.7%)	(84.7%)	
Echo pattern				0.314
Hypoechoic	251	55	196	
	(90.6%)	(88.7%)	(91.2%)	
Isoechoic	11	2	9	
	(3.9%)	(3.2%)	(4.2%)	
Complex cystic	3	0	3	
	(1.1%)	0	(1.4%)	
Heterogeneous	12	5	7	
	(4.4%)	(8.1%)	(3.2%)	
SWS_SD_ (m/s)	0.74 ± 0.73	1.74 ± 0.81	0.45 ± 0.36	<0.001*
E-mean (kPa)	31.2 ± 26.9	62.2 ± 32.3	22.2 ± 16.9	<0.001*
E_SD_ (kPa)	14.9 ± 16.1	36.9 ± 16.8	8.5 ± 8.3	<0.001*
SWS-mean (m/s)	2.91 ± 1.29	4.28 ± 1.51	2.51 ± 0.88	<0.001*

*Indicates a significant difference.

Data are shown as means ± standard deviations; SWS_SD_ = standard deviation of shear wave speed; E-mean = the mean elasticity; E_SD_ = standard deviation of elasticity; SWS-mean = the mean shear wave speed; Changed posterior features include enhancement, shadowing and combined pattern.

### Diagnostic performances of shear wave elastography

The values of E-mean, E_SD_, SWS-mean and SWS_SD_ in malignant breast lesions were significantly higher than those of benign lesions (Table 2). Using quantitative parameters of shear wave elastography, breast lesions with values greater than or equal to the cut-off values were considered as malignancy whereas the remaining breast lesions were classified as benign. Compared with other quantitative shear wave parameters, SWS_SD_ had the highest AUROC value of 0.896 (95% CI: 0.840, 0.953) with the optimal cut-off value at 1.14 m/s. SWS_SD_ had a sensitivity of 75.8%, specificity of 95.8%, accuracy of 91.3%, PPV of 83.9%, and NPV of 93.2% (Table 3).

**Table 3:** The Diagnostic Performances of all the SWE Methods.

Variables	Cut-off value	Sensitivity (%)	Specificity (%)	Accuracy (%)	PPV (%)	NPV (%)	AUC	95% CI
E-mean (kPa)	39.6	77.4	87.9	85.5	64.8	93.1	0.844	0.773–0.914
		(48/62)	(189/215)	(237/277)	(48/74)	(189/203)		
SWS-mean (m/s)	3.53	75.8	87.9	85.2	64.4	92.6	0.825	0.752–0.898
		(47/62)	(189/215)	(236/277)	(47/73)	(189/204)		
E_SD_ (kPa)	19.9	82.2	91.6	89.5	73.9	94.7	0.894	0.832–0.955
		(51/62)	(197/215)	(248/277)	(51/69)	(197/208)		
SWS_SD_ (m/s)	1.14	75.8*	95.8*	91.3	83.9^※^	93.2	0.896	0.840–0.953
		(47/62)	(206/215)	(253/277)	(47/56)	(206/221)		
SWATC display		91.9^★^	90.7	90.9	74.0	97.5	0.913	0.868–0.958
		(57/62)	(195/215)	(252/277)	(57/77)	(195/200)		
SWATC display+ SWS_SD_		93.5*	89.3*	90.2	71.6	97.9	0.914	0.871–0.957
		(58/62)	(192/215)	(250/277)	(58/81)	(192/196)		

SWS_SD_ = standard deviation of shear wave speed; E-mean = the mean elasticity; E_SD_ = standard deviation of elasticity; SWS-mean = the mean shear wave speed; PPV = positive predictive value; NPV = negative predictive value; CI = confidence interval; AUC = area under characteristic curve;

SWATC = shear wave arrival time contour.

*There are statistically significant difference between sensitivity and specificity of SWS_SD_ and SW arrival time contour+SWS_SD._(P < 0.05)

^★^SW arrival time contour display showed higher sensitivity compared with E-mean, SWS-mean, and SWS_SD_ with statistically significant difference. (P < 0.05)

^※^SWS_SD_ had higher PPV compared with E-mean, SWS-mean, E_SD_, SWATC display and SW arrival time contour+ SWS_SD_ with statistically significant difference (P < 0.05).

Breast lesions with shear wave propagation quality score of 1 (low quality) were classified as malignant whereas those with score of 2 (high quality) were classified as benign. Among the 277 lesions, 77 had a score of 1 (low quality) and 200 had a score of 2 (high quality). There were 57 (74%) malignant and 20 (26%) benign lesions in the low image quality group, and 5 (2.5%) malignant and 195 (97.5%) benign lesions in the high image quality group.

The AUROC value of shear wave propagation quality score based on arrival time contour display was 0.913 (95% CI: 0.868–0.958), with a sensitivity of 91.9%, a specificity of 90.7%, a PPV of 74.0% and an NPV of 97.5%. Those with diameters greater than 15mm, shear wave propagation quality score had a sensitivity of 100%, specificity of 82.7%, accuracy of 88.4%, PPV of 75%, and NPV of 100%. We also investigated if diagnosis performance can be improved by combining shear wave propagation quality score and SWS_SD_. In this method, breast lesions with SWS_SD_ greater than or equal to the cut-off value were classified as malignant. In addition, lesions with shear wave quality score of 1 were classified as malignant regardless of SWS_SD_. All remaining lesions were classified as benign. Compared with SWS_SD_ alone, the sensitivity increased from 75.8% to 93.5% (P < 0.001) while specificity decreased from 95.8% to 89.3% (P < 0.05) by combining shear wave propagation quality score and SWS_SD_.

### Factors associated with the quality of shear wave propagation

We investigated the following possible factors: patient age, tumor malignancy, lesion diameter, lesion depth, shape, orientation, margin, SWS_SD_, E_SD_, E-mean, SWS-mean and posterior feature. In univariate analysis, larger lesion size, higher value of SWS_SD_, E_SD_, E-mean, SWS-mean, deeper lesion depth, and malignancy were significantly associated with lower QSWP. Regular shape, parallel orientation, unchanged posterior features, and circumscribed margin were more commonly found in images with higher QSWP (all *P* < 0.05). Binary logistic regression analysis showed that SWS_SD_ was the most important factor associated with the QSWP, with an odds ratio (OR) of 86.05 (95% CI: 2.947–2513; P < 0.05), followed by tumor malignancy (OR: 22.53; 95% CI: 4.028–126.1) and the depth of lesion (OR: 6.19; 95% CI: 1.811–21.22).

## Discussion

Shear wave elastography provides quantitative value of lesion stiffness in unit of kilopascals or meters per second, which has shown promise in improving the diagnosis performance of breast lesion imaging^13, 14^. Studies have shown that selection of high-quality shear wave images is important because image quality substantially influences the performance for tumor diagnosis^15^. The SWATC display provides direct visual feedback for the user to assess the reliability of shear wave elastography. Unreliable shear wave measurements may result from technical reasons such as patient motion and transducer motion. For breast imaging, shear wave images with minimal probe and patient motion usually can be obtained by experienced sonographers or radiologists. After excluding these technical factors, if a solid lesion is not color coded or has low quality of shear wave propagation, it has a high probability of being malignant. In our study, SWATC display was used to determine if a lesion was benign or malignant. Compared with E-mean, E_SD_, SWS-mean, and SWS_SD_, the sensitivity of SWATC display score was significantly higher, while specificity was similar. The AUROC value of the SWATC display score was highest among these elastography parameters. It seems that the application of SWATC display score in the diagnosis of breast lesions is promising. Compared with SWS_SD_ alone, the sensitivity of combined SWS_SD_ and SWATC display score increased from 75.8% to 93.5% while specificity decreased from 95.8% to 89.3%, which is consistent with previous studies^8^. In our study, SWATC display score had a NPV of 100% for the lesions which are greater than 15mm. When the breast mass is greater than 15 mm in diameter with shear wave quality score of 2, we may not do puncture but follow up in clinical practice. With the help of SWATC display, it may also be possible to intelligently select regions of reliable shear wave measurements for lesion characterization to improve diagnostic accuracy.

Previous studies have reported that invalid shear wave speed measurements or low QSWP were more frequently found in malignant breast lesions^8, 16^. To use shear wave measurement quality as diagnostic information, it is important to study confounding factors associated with shear wave measurement quality. In this study, we investigated various patient and lesion factors that can influence the QSWP. We found that lesion size, value of E_SD_, SWS_SD_, and E-mean, lesion depth, malignancy, shape, orientation, posterior features, margin, and calcification were significantly correlated with QSWP. Results of binary logistic regression analysis showed that SWS_SD_ was the most important factor associated with the QSWP, followed by malignancy and the depth of lesion. According to the depth of the lesion, we divided it into two subgroups (Group 1, ≤15 mm; Group 2, >15 mm) in the present study. In 187 breast lesions with the depth less than or equal to 15 mm, 85.6% (160/187) breast lesions had high QSWP, 14.4% (27/187) breast lesions had low QSWP. However, in 90 breast lesions with the depth more than 15 mm, 44.4% (40/90) breast lesions had high QSWP, 55.6% (50/90) breast lesions had low QSWP. In a study by Chang *et al*.^17^, they found that lesion depth significantly correlates with image quality for strain elastography. For shear wave measurements based on acoustic radiation force, the penetration of linear transducers is typically less than 4.5 cm. And the depth of lesion would affect the QSWP for breast lesions^18–20^. This is probably due to tissue attenuation of ultrasound: as the depth increases, the push pulse is attenuated more, which leads to lower shear wave amplitude. In addition, ultrasound detection pulses attenuate more with increased depth, leading to less reliable detection. As a result, the reliability of shear wave measurements generally decreases with depth. Therefore, the effects of lesion depth should be properly accounted for in order to improve the accuracy of lesion diagnosis using shear wave propagation quality.

Tumor malignancy was identified as another factor associated with low quality of shear wave propagation (OR:22.53). In our study, low QSWP was more commonly found in malignant breast lesions. Tissue inhomogeneity might be one reason for low QSWP in malignancy. Malignant breast lesions are histologically heterogeneous due to lymphocytic infiltrates and necrosis^21^, whereas benign breast lesions generally have a more uniform pathological structure. Tissue inhomogeneity can distort shear wave propagation contour lines and lead to a low QSWP score. The heterogeneity of breast lesions can also be assessed by SWS_SD_^22^: higher value of SWS_SD_ indicates higher degree of heterogeneity. In this study, SWS_SD_ was found to be associated with QSWP.

There were some limitations in the study. First, the intra-operator and inter-operator consistency of quantitative shear wave speed measurements and QSWP were not assessed in our study. Previous studies showed that shear wave measurements were highly reproducible for assessing breast lesions and thus observer variability is not expected to have large influence on this study^23, 24^. Second, only patients and lesions factors were evaluated for association with shear wave propagation quality, while equipment related factors (such as thermal noise, finite signal bandwidth, and the geometric spreading and absorbing of shear waves) were no studied^25^. Third, the retrospective nature of the study could not avoid selection bias and future prospective study is needed. Finally, this study was single-center study, further prospective study with multicenter collaborations is needed to verify these results.

In conclusion, SWATC display showed promising diagnostic performance and may be used as a reference to place the region of interest (ROI) for shear wave speed measurement and characterization of breast lesions. SWS_SD_ was the most important factors associated with QSWP, followed by tumor malignancy and the depth of lesion.
